# Jasmonate-responsive expression of paclitaxel biosynthesis genes in *Taxus cuspidata* cultured cells is negatively regulated by the bHLH transcription factors TcJAMYC1, TcJAMYC2, and TcJAMYC4

**DOI:** 10.3389/fpls.2015.00115

**Published:** 2015-02-26

**Authors:** Sangram K. Lenka, N. Ezekiel Nims, Kham Vongpaseuth, Rosemary A. Boshar, Susan C. Roberts, Elsbeth L. Walker

**Affiliations:** ^1^Department of Biology, University of MassachusettsAmherst, MA, USA; ^2^Plant Biology Graduate Program, University of MassachusettsAmherst, MA, USA; ^3^Department of Chemical Engineering, University of MassachusettsAmherst, MA, USA

**Keywords:** *Taxus cuspidata*, paclitaxel, methyl jasmonate, E-box, JA-MYC, MYC2

## Abstract

*Taxus* cell suspension culture is a sustainable technology for the industrial production of paclitaxel (Taxol®), a highly modified diterpene anti-cancer agent. The methyl jasmonate (MJ)-mediated paclitaxel biosynthetic pathway is not fully characterized, making metabolic engineering efforts difficult. Here, promoters of seven genes (TASY, T5αH, DBAT, DBBT, PAM, BAPT, and DBTNBT), encoding enzymes of the paclitaxel biosynthetic pathway were isolated and used to drive MJ-inducible expression of a GUS reporter construct in transiently transformed *Taxus* cells, showing that elicitation of paclitaxel production by MJ is regulated at least in part at the level of transcription. The paclitaxel biosynthetic pathway promoters contained a large number of E-box sites (CANNTG), similar to the binding sites for the key MJ-inducible transcription factor AtMYC2 from *Arabidopsis thaliana*. Three MJ-inducible MYC transcription factors similar to AtMYC2 (TcJAMYC1, TcJAMYC2, and TcJAMYC4) were identified in *Taxus*. Transcriptional regulation of paclitaxel biosynthetic pathway promoters by transient over expression of TcJAMYC transcription factors indicated a negative rather than positive regulatory role of TcJAMYCs on paclitaxel biosynthetic gene expression.

## Introduction

**Figure 1:**
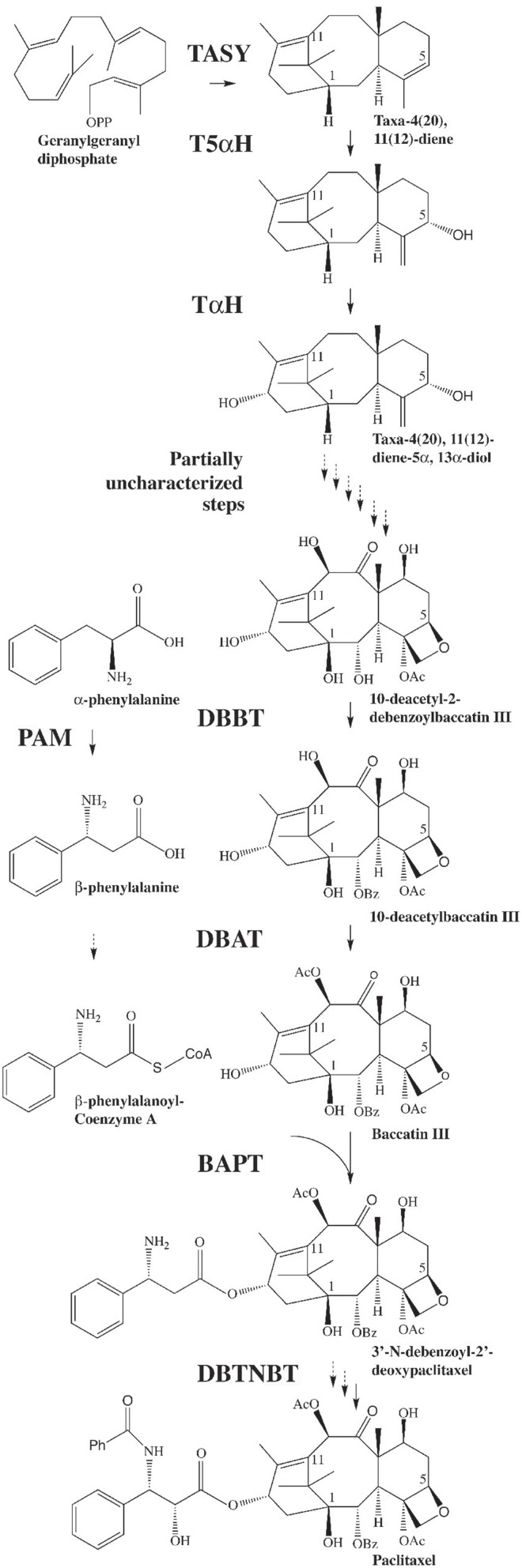
**The biosynthetic pathway leading to paclitaxel**. GGPP is synthesized by geranylgeranyl diphosphate synthase (GGPPS). GGPP is then converted to taxadiene by taxadiene synthase (TASY) (Wildung and Croteau, 1996) and then to taxadien-5α-ol by taxadien-5α-ol hydroxylase (T5α H) (Jennewein et al., 2004a). Several steps leading from the diol intermediate to functionalized taxanes, including the formation of the oxetane ring, are unknown. Taxane 2α-O-benzoyltransferase (DBBT) (Walker and Croteau, 2000b) produces 10-deacetylbaccatin III (10-DAB). 10-DAB is then converted to baccatin III by 10-deacetylbaccatin III-10-O-acetyltransferase (DBAT) (Walker and Croteau, 2000a). Baccatin III: 3-amino, 3-phenylpropanoyltransferase (BAPT) ligates the side chain [derived from phenylalanine via phenylalanine aminomutase (PAM) (Jennewein et al., 2004b)] to produce 3′-N-debenzoyl-2-deoxypaclitaxel (Walker et al., 2002a). An unknown P450-mediated hydroxylation of the side chain forms 3′-N-debenzoyl paclitaxel. 3′-N-debenzoyl-2-deoxypaclitaxel-N-benzoyltransferase (DBTNBT) then produces paclitaxel by benzoylation of the side chain (Walker et al., 2002b).

Paclitaxel (Taxol®; Figure 1) is a diterpene derived from plants in the genus *Taxus*. Taxol triggers anti-mitotic and cytotoxic activity by disrupting normal tubulin dynamics leading to dysfunction of microtubules (Schiff et al., 1979). Clinical application of Taxol® has been approved by the US Food and Drug Administration for several types of cancer treatment (www.fda.gov). Paclitaxel is also being used in arterial stents to inhibit scar tissue formation after implant (Bajaj and Garratt, 2010), thus the demand for this important compound is expected to increase. Paclitaxel constitutes only 0.01–0.03% of the dry weight of the bark of *Taxus* and total synthesis comprises several steps, and is therefore low yielding (Fu et al., 2009). Currently paclitaxel and its precursor are primarily derived from the needles of yew plants as well as *Taxus* suspension cell cultures (Frense, 2007; Vongpaseuth and Roberts, 2007; Kolewe et al., 2008; Ajikumar et al., 2010; Flores-Bustamante et al., 2010). The biosynthetic pathway leading to paclitaxel has been only partially elucidated (Croteau et al., 2006; Ketchum et al., 2007; Long et al., 2008) (Figure 1), and improved understanding of the four or five undefined pathway steps as well as the overall regulation of paclitaxel synthesis are needed in order to enable bioengineering approaches. This will allow enhanced production of paclitaxel and potentially may allow production of novel bioactive taxanes in plant cells.

*Taxus* suspension cultures are commonly elicited using exogenous methyl jasmonate (MJ) to produce paclitaxel (Yukimune et al., 1996). The transcript levels of the known paclitaxel biosynthetic pathway genes in *Taxus* suspension cultures increase within 6 h after elicitation. Transcript levels peak at 12–18 h and then taper off to basal levels within 30 h. Accumulation of the taxane intermediates 10-deacetyl baccatin III and baccatin III (Figure 1) parallels transcript profiles, but with a 12–18 h lag. However, accumulation of paclitaxel occurs much later (3–6 days after elicitation), long after steady state transcript levels of the known pathway genes have returned to normal (Nims et al., 2006; Lenka et al., 2012). The mechanism(s) underlying the delayed correlation between steady state mRNA levels and accumulation of the desired metabolite paclitaxel are not understood. Still, the coordinated expression of the known paclitaxel pathway genes suggests that these genes could be under the control of a common regulatory regime. Generally, MJ application triggers profound transcriptional reprogramming in plant cells to modulate control machineries of a wide range of metabolite biosynthesis by interplay of both positive and negative regulators (Fonseca et al., 2009). Better understanding of the events between MJ application and metabolite accumulation will be useful for developing strategies to enhance production of taxanes in cultured cells.

Although MJ elicits a wide range of species-specific and structurally diverse secondary metabolic pathways of different biochemical origins, it appears that the basic MJ signaling machinery may be conserved in plants of different phylogenetic lineages (De Geyter et al., 2012). Key regulators belonging to the bHLH class of proteins regulating specialized Jasmonic acid (JA) mediated metabolite production have been identified in *Catharanthus roseus, Arabidopsis thaliana, Nicotiana tabacum*, and *Nicotiana benthamiana* (van der Fits and Memelink, 2000, 2001; van der Fits et al., 2000; Siberil et al., 2001; Chatel et al., 2003; Dombrecht et al., 2007; Gigolashvili et al., 2007a,b, 2008; Pre et al., 2008; Todd et al., 2010; Qi et al., 2011; Zhang et al., 2011, 2012). MYC2 can act as a positive regulator for several transcription factor (TF) genes and for genes encoding flavonoid biosynthesis pathway, but can also be a negative regulator, for example, of genes involved in indole glucosinolate synthesis (Dombrecht et al., 2007). In the absence of MJ, MYC2 action is repressed by JAZ proteins by forming repressor complexes with a group of other proteins including Novel Interactor of JAZ (NINJA) and TOPLESS (Browse, 2009; Chini et al., 2009). MJ elicitation leads to JAZ degradation by the SCFCOI1–ubiquitin–proteasome pathway releasing MYC2 from the repressor complex. The unbound MYC2 regulates target MJ-responsive genes by binding to E-boxes present in their promoters (Browse, 2009; Chini et al., 2009). The JA signaling mechanism also oscillates through a negative feedback loop involving MYC2 and JAZ proteins, in which JAZ blocks MYC2 activity at the protein level and MYC2 transcriptionally induces JAZ expression (Chini et al., 2007). Hence, upon MYC2 overexpression, MYC2 activity would be influenced by the relative abundance of the activator MYC2 and the JAZ repressor proteins.

In this study we have investigated the role of three MJ-regulated bHLH TFs in *Taxus cuspidata*. These bHLH potential regulators are named JAMYC (TcJAMYC1, TcJAMYC2, and TcJAMYC4), based on their similarity to Arabidopsis MYC2, which has also been called JAMYC (Lorenzo et al., 2004). The similarity in sequence and expression between TcJAMYC(s) and the well-characterized AtMYC2 suggests a conserved response to MJ despite significant divergence between the gymnosperm and angiosperm lineages. The results presented here suggest that TcJAMYC(s) are regulators of MJ-mediated expression of taxane biosynthetic genes in *Taxus* cell cultures, but that they function as negative rather than positive regulators of these genes.

## Materials and methods

### Cell culture, MJ elicitation, and transient transformation

Taxol producing *Taxus cuspidata* P991 suspension cell cultures were used for all experiments and are grown as described previously (Nims et al., 2006). For elicitation, 40 μl of 100% methyl jasmonate was added to 960 μl of 50% ethanol, the modified protocol used as described by (Patil et al., 2013). This solution was then filtered through a 0.2 μm PVDF filter. Cells were either elicited with MJ (dissolved in 50% EtOH) at a 100 μm final concentration or mock-elicited by adding 50% of equal volume EtOH. After 3 h, both batches of cells (~0.5 g) were transferred onto B5-agar plates, spread out in a circle in the center of the plate with a diameter of about 3 cm, and gently pressed into the agar to immobilize them. Within 2 h, the cells were transformed by bombardment using a PDS-1000 (Bio-RAD, Hercules, CA). Preparation of gold microcarriers was performed as described (Vongpaseuth et al., 2007). A minimum of three replicates of each experiment were performed. Forty-eight hours between bombardment and assay was provided to allow for optimal expression of the reporter genes.

### GUS and LUC assays

Transformed cells were ground in lysis buffer (1 ml; 100 mM KHPO_4_ pH 7.8 with 0.2% Triton X-100) using 3.2 mm chrome steel beads for 10 min at 30 Hz in a Retsch® MM 400 mixer mill (Retsch Inc., Irvine, California). The cell lysate was incubated on ice for 5 min, and the debris was removed by two rounds of centrifugation for 10 min at 16,000 × *g*. Luciferase and GUS activity of 10 μl of the cell lysate was measured by using Applied Biosystems' Tropix® Dual-Light® assay (Applied Biosystems, Foster City, CA) as per the manufacturer's instructions in a SpectraMax® M5 multi-mode microplate reader (Molecular Devices, Sunnyvale, CA).

### Cloning of paclitaxel biosynthetic gene promoters

The upstream flanking regions of the biosynthetic pathway genes were cloned using inverse-PCR (Ochman et al., 1988) based on the cDNA sequences of DBBT (AF297618), DBAT (AF193765), PAM (AY582743), BAPT (AY563630), and DBTNBT (AF466397). *TASY* and *T5αH* promoters were cloned using PCR based on the sequence information of EF153471 and EF153470, respectively. PCR products were cloned into pDESTG221 to form translational fusions to GUS with 5–40 N-terminal amino acid residues of the pathway biosynthetic gene prior to the fusion point with GUS. *cis-*element analysis of these pathway promoters were performed using PLACE database (http://www.dna.affrc.go.jp/PLACE/) (Higo et al., 1999).

### Vector construction

A vector, pDESTG221, was constructed by modifying pPZP221 (Hajdukiewicz et al., 1994) to contain a Gateway recombination cassette B (Invitrogen, Carlsbad, CA), GUS reporter gene (Jefferson et al., 1987) and NOS terminator. The *Taxus* promoter DNA fragments were subcloned into pCR8/GW/TOPO vector and transferred to pDESTG221 using Gateway LR Clonase II (Invitrogen, Carlsbad, CA), creating translational fusions to the GUS reporter gene. The TcJAMYC TFs were amplified from the first-strand cDNA of MJ-elicited *Taxus cuspidata* suspension cell culture and ligated in between the CaMV35S promoter and a NOS 3′ sequence. The 35S:GUS construct was taken from pBI121:GUS and placed into the multiple cloning site of pPZP221 using EcoRI and BamHI restriction sites. The 35S:LUC construct was ligated into pTZ19u.

### Degenerate primer amplification of TcJAMYC1

The conserved bHLH domain in the JAMYC proteins from *Solanum tuberosum* (AJ630505) (Boter et al., 2004) and *Arabidopsis* (At1g32640) (Abe et al., 1997) was used to design degenerate primers with the Consensus-Degenerate Hybrid Oligonucleotide Primers (CODE-HOP) program (Rose et al., 2003). Five primers were chosen, and two (Forward primer oDEGmyc.1 GAGAAGAACCTCTGAATCATGTTGARGCNGARMG; Reverse primer oDEGmyc.3 CAGCTTACATTTCAGTTCATTAATATAAGAAATNGCRTCNCC) produced a PCR product that was then used to screen a cDNA library by hybridization.

### Cloning of TcJAMYC1, TcJAMYC2, and TcJAMYC4

Total RNA was extracted from two grams of *Taxus cuspidata* cell culture line P991, by guanidium isothiocyanate and cesium chloride gradient ultracentrifugation at 104,000 × *g* for 18 h, followed by phenol-chloroform extraction. Poly-A RNA (5 μg) was obtained from 1 mg total RNA using Poly-A Purist Mag-Kit (Ambion, Austin, TX). cDNA construction and cloning was performed using the ZAP Express cDNA Synthesis Kit (Agilent Technologies, Cedar Creek, TX). Plaques (1 × 10^6^) from the primary library were screened using the *Taxus* bHLH fragment obtained by PCR. A 2.5 kb cDNA clone was isolated and sequenced, but the 5′-end was truncated. 5′-Rapid Amplification of cDNA Ends (RACE) was performed using RLM-RACE kit (Ambion, Austin, TX). The full-length cDNA of TcJAMYC1 (FJ608574) was cloned by PCR using the 5′-sequence obtained by RACE, and this product was cloned and sequenced. TcJAMYC2 (JX519289) and TcJAMYC4 (JX519290) were cloned by using sequence specific primers designed by mining an in-house 454 GS FLX sequencing dataset.

### Phylogenetic analysis

Multiple sequences alignments of the full length bHLH proteins were performed using ClustalW with default settings. Bootstrap method for estimating the standard error is used to plot the phylogram. The unrooted phylogenic tree was generated using MEGA5 (Tamura et al., 2011) by the Neighbor-Joining algorithm and values at branch-nodes are percentages of 1000 bootstrap repetitions. Also included in the analysis are the previously reported bHLH TFs recruited by JA signaling to induce secondary metabolite biosynthesis such as AtMYC2 (NM102998), CrMYC2 (AF283507), NtMYC2a (HM466974), NtMYC2b (HM466975), NbNbbHLH1 (GQ859152), NbNbbHLH2 (GQ859153), AtGL3 (NM148067), AtEGL3 (NM105042), AtTT8 (NM117050), AtbHLH28 (AT5G46830), AtMYC3 (At5G46760), and AtMYC4 (AT4G17880).

### TcJAMYC1 protein purification

The TcJAMYC1 cDNA was recombined into pDEST17 (Invitrogen), an *E. coli* expression vector containing an N-terminal 6X His tag for affinity purification on a Nickel agarose column (Qiagen, Valencia, CA). The Rosetta 2(DE3) pLysS (EMD biosciences, Gibbstown, NJ) strain of *E. coli* containing this construct was grown to late log phase (OD_600_ = 0.8), induced with 1 mM IPTG, and then incubated with shaking for four more hours at 37°C. Cells were pelleted by centrifugation at 4400 × *g*, resuspended in 50 mM Tris-Cl pH 6.8, 20 mM β-mercaptoethanol, 2% SDS, 10% glycerol, and 10 mM imidazole, and 50 μl DNase1 (10 mg/ml), then incubated on ice for 30 min. Debris was removed by centrifugation at 17,000 × *g* for 20 min, and the TcJAMYC1 protein was bound to Ni-NTA resin (Qiagen, Valencia, CA). The resin was washed with buffer containing 250 mM NaCl, 50 mM Tris-Cl pH 6.8, 20 mM imidazole, and eluted with buffer containing 250 mM NaCl, 50 mM Tris-Cl pH 6.8, 300 mM imidazole. The eluted protein was further purified using a Centricon YM-3 centrifugal filter device (Millipore, Danvers, MA) and brought to a final protein concentration of 25 ng/μl in 50% glycerol.

### Electrophoretic mobility shift assays (EMSA)

Oligonucleotide probes (see Table 1) contained a six nucleotide E-box at the center of a 22 bp sequence. A four-nucleotide 5′-overhang was included in each double stranded probe to allow for ^32^P-labeling. Labeling reactions contained 100 mM Tris-HCl, 50 mM NaCl, 10 mM MgCl_2_, 0.025 % Triton X-100, pH 7.5, 2.2 mM of each dTTP, dATP, and dGTP and 22 μm double stranded oligo in a 23 μl total volume with 5 U of Klenow large fragment DNA polymerase (New England Biolabs, Ipswich, MA). Unincorporated nucleotides were removed using a 2 ml Sephadex G-25 (Sigma, St. Louis, MO) column. For each EMSA reaction, 175 fmol of double stranded oligonucleotide were used (labeling reaction diluted 1:125). EMSA buffer consisted of 20 mM HEPES-KOH pH 7.9, 20% glycerol, 0.2 mM EDTA, 100 mM KCl, 0.5 mM PMSF, and 1 mM DTT, 15 mM MgCl_2_, and 5 μg BSA. For standard reactions, 2 μl of TcJAMYC1 protein was used and the final volume of the reactions was 20 μl. Native polyacrylamide gel (7% acrylamide (29:1), 1% glycerol, 0.5X TBE) electrophoresis was used to separate the DNA probe that was bound by the protein and the free DNA probe. Gels were run at 4°C for 1.5 h at 82 V after being pre-run for 30 min.

**Table 1:** **Oligonucleotide probes used for EMSA**.

**CATGTG:**
TAGCGCATCGAT**CATGTG**ATCGATCG
CGTAGCTA**GTACAC**TAGCTAGCATGC
**CACGTG:**
TAGCATCGATCG**CACGTG**ATCGATCG
TAGCTAGC**GTGCAC**TAGCTAGCATGC
**CAAGTG:**
TAGCGCATCGAT**CAAGTG**ATCGATCG
CGTAGCTA**GTTCAC**TAGCTAGCATGC
**CAATTG:**
TAGCGCATCGAT**CAATTG**ATCGATCG
CGTAGCTA**GTTAAC**TAGCTAGCATGC
**CAACTG:**
TAGCGCATCGAT**CAACTG**ATCGATCG
CGTAGCTA**GTTGAC**TAGCTAGCATGC
**CACCTG:**
TAGCGCATCGAT**CACCTG**ATCGATCG
CGTAGCTA**GTGGAC**TAGCTAGCATGC
**CATCTG:**
TAGCGCATCGAT**CATCTG**ATCGATCG
CGTAGCTA**GTAGAC**TAGCTAGCATGC
**CATTTG:**
TAGCGCATCGAT**CATTTG**ATCGATCG
CGTAGCTA**GTAAAC**TAGCTAGCATGC
**CATATG:**
TAGCGCATCGAT**CATATG**ATCGATCG
CGTAGCTA**GTATAC**TAGCTAGCATGC
**Mutated:**
TAGCGCATCGAT**AAGCCT**ATCGATCG
CGTAGCTA**TTCGGA**TAGCTAGCATGC

*26mer oligonucleotides were used as double stranded DNA in the EMSA. Putative E-box elements are in bold face. The mutated probe does not contain the CANNTG sequence that defines the generic E-box sequence. Out of nine E-boxes that are found in the pathway promoters, eight are represented*.

### Sub-cellular localization of TcJAMYC1

A C-terminal translational fusion was made by cloning in frame TcJAMYC1 cDNA and soluble modified GFP (smGFP) into the psmGFP vector (CD3-326; Arabidopsis Biological Resource Center) under the control of a CaMV35S promoter. The 35S::*TcJAMYC1::GFP* fusion construct was then transiently expressed in intact Arabidopsis mesophyll protoplasts as per the tape-Arabidopsis sandwich method (Wu et al., 2009). Protoplasts were imaged by a Zeiss 510 Meta laser scanning confocal microscope (Nikon) with excitation at 488 nm, and the fluorescence emission signal was recovered between 520 and 550 nm.

## Results

### MJ induces paclitaxel pathway gene promoters

**Figure 2:**
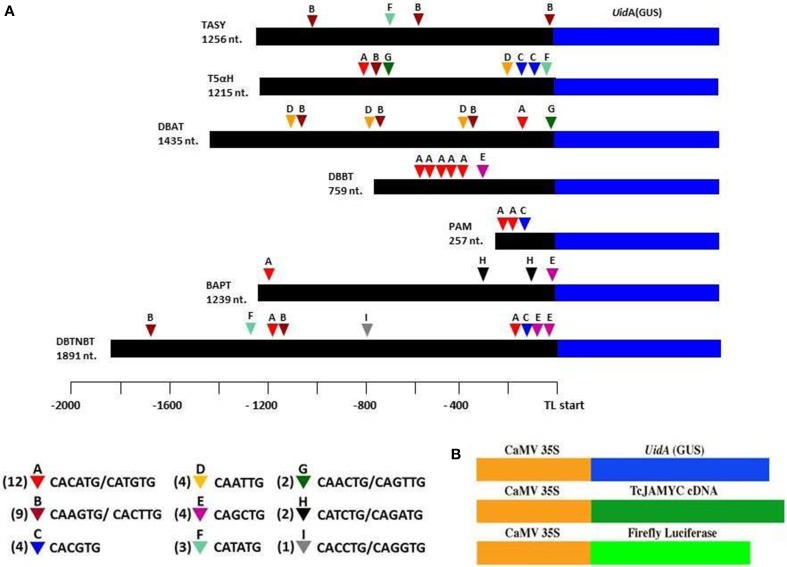
**The pathway promoters cloned using inverse-PCR and the locations of the various E-box elements found in all seven gene promoter sequences**. **(A)** Numbers next to the triangles indicate the frequency of that E-box element. Promoter regions were translationally fused to GUS so that 5–40 codons after the ATG of the paclitaxel pathway gene are encoded as an N-terminal extension on the GUS protein. **(B)** The GUS TcJAMYC(s) full-length cDNA, and firefly luciferase (LUC) genes, all driven by the Cauliflower Mosaic Virus (CaMV) 35S promoter, that were used in bombardment assays.

**Figure 3:**
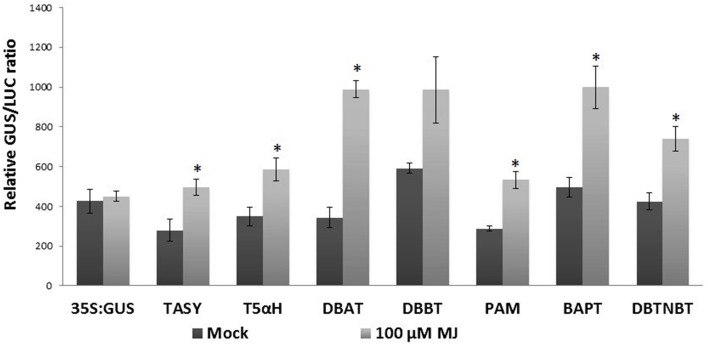
**Pathway promoter activation by MJ**. *Taxus* suspension cultures were plated onto B5 agar plates and bombarded with the promoter:GUS reporter constructs. 35S:GUS was also bombarded as control. Forty-eight hours was allowed between bombardment and assaying the GUS activity. Results are presented as a GUS/LUC ratio. *n* = 3 in all samples, error bars are SE (^*^p < 0.05, *t*-test).

Transcript profiling studies in *Taxus* cultured cells previously demonstrated the MJ-induced mRNA accumulation of the paclitaxel biosynthetic pathway genes (hereafter referred to as “pathway genes”) (Nims et al., 2006; Lenka et al., 2012). To investigate MJ-responsive promoter activity of pathway genes, promoters of seven known pathway genes *viz*. taxadiene synthase (*TASY*; JF338876), taxadien-5α-ol hydroxylase (*T5αH*; JF338877), taxane 2α-O-benzoyltransferase (*DBBT*; JF338880), 10-deacetylbaccatin III-10-O-acetyltransferase (*DBAT;* FJ603641), phenylalanine aminomutase (*PAM;* FJ603642), baccatin III: 3-amino, 3-phenylpropanoyltransferase (*BAPT;* JF338879) and 3′-N-debenzoyl-2-deoxypaclitaxel-N-benzoyltransferase (*DBTNBT;* FJ603644) were cloned. Constructs containing a β-glucuronidase (GUS) reporter gene driven by each of these promoters were made (Figure 2A), and were delivered into the *Taxus cuspidata* P991 culture cells for transient reporter gene expression by particle bombardment. The cells were either mock-elicited or elicited with MJ at a final concentration of 100 μm. *35S:LUC* was co-bombarded in all experiments as control for variable transformation efficiency (Figure 2B). Reporter gene activity is presented as a GUS/LUC ratio to control for transformation efficiency between samples. For each pathway gene promoter construct, GUS enzyme activity was at least 1.5-fold higher in MJ-elicited cells compare to the control (Figure 3). Thus, we concluded that the promoters of the pathway genes are activated by MJ. This result, in combination with increased amount of steady state mRNA for the endogenous genes after MJ elicitation (Nims et al., 2006; Lenka et al., 2012; Li et al., 2012b), implies that MJ treatment causes increased transcription of pathway genes.

### Promoter analysis

Since the pathway gene promoters were activated upon MJ-elicitation and since all the pathway genes respond in a uniform time course (Nims et al., 2006; Li et al., 2012b), there could be an MJ-responsive transcriptional regulatory mechanism that influences these promoters. Considering this, an *in silico* analysis of the pathway promoters was performed using PLACE (http://www.dna.affrc.go.jp/PLACE/) (Higo et al., 1999). This analysis revealed that there are 37 E-boxes (CANNTG) found in the 8052 nt of the collective pathway gene promoter sequence that we cloned (Figure 2A). A disproportionately large number of potential E-box sites are found in the promoters of the pathway genes, since only 31 E-boxes would be expected to occur at random in a sequence of this length. E-boxes have been found in defense gene promoters in other plants (Kim et al., 1992). bHLH proteins typically bind to these E-box nucleotide motifs (Qian et al., 2007). For example, the *Arabidopsis* bHLH MYC2 protein preferentially binds to the E-box sequence CACGTG. Furthermore, E-boxes are commonly found on the promoters of genes that respond to MJ (Dombrecht et al., 2007; Montiel et al., 2011). The *Solanum* JAMYC2 binds a related, “T/G-box” sequence (AACGTG) on the proteinase inhibitor promoter (Boter et al., 2004). The T/G-box was found in only four of the *Taxus* gene promoters (DBBT (twice), TASY, DBAT, and DBTNBT). Hence, the over-representation of E-box sites on the cloned promoters suggests the MJ-mediated transcriptional regulation of pathway promoters could be imparted by jasmonate-responsive bHLH MYC proteins.

### Cloning TcJAMYC1, TcJAMYC2, and TcJAMYC4

**Figure 4:**
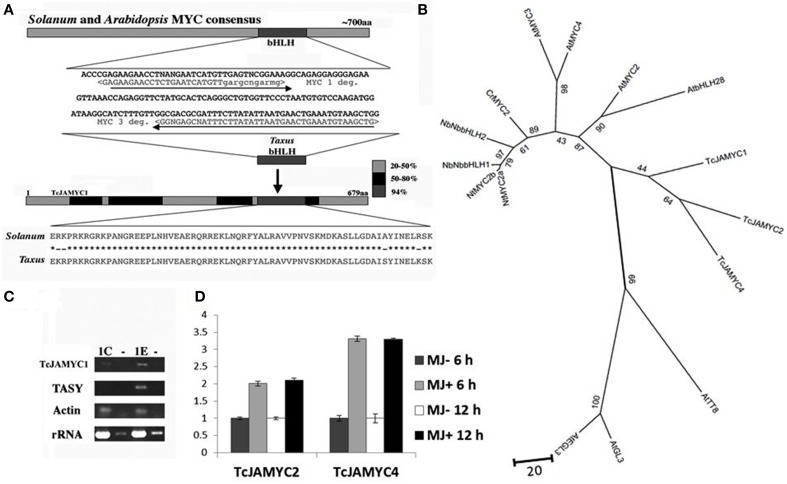
**Cloning of TcJAMYC1**. **(A)** Degenerate primers were designed from the conserved bHLH region in order to amplify a *Taxus* DNA fragment. This fragment was used to obtain a full-length cDNA. **(B)** The relationship between the three TcJAMYCs and 12 other known bHLH protein sequences. The tree was created by using 12 known bHLH proteins recruited by JA signaling for enhanced production of secondary metabolite biosynthesis in other plants along with the TcJAMYCs protein. This is an unrooted Neighbor-Joining phylogram and values at branch-nodes are percentages of 1000 bootstrap repetitions. **(C)** RT-PCR analysis of *TcJAMYC1*, Taxadiene synthase (TASY, AY424738) *rRNA* (AF259290), and *actin* (derived from *P. contorta* actin; Genbank M36171.1) after 1 h of MJ elicitation. Both *rRNA* and *actin* fragments were amplified as internal controls. The control (mock elicited) cells are labeled 1C and the MJ-elicited cells are labeled 1E. The lanes labeled (–) represent the results of amplification of RNA without reverse transcription, as a control for contaminating genomic DNA in our RNA preparations. (**D**) qRT-PCR analysis of *TcJAMYC2* and *TcJAMYC4* after 6 and 12 h of MJ elicitation. Induction values for target genes normalized to the endogenous controls *Taxus* actin (JF735995). Error bars are SD.

The well-conserved bHLH sequences from *Arabidopsis* MYC2 (At1g32640) and *Solanum* JAMYC10 (AJ630506) were used to design degenerate primers with the CODE-HOP program (Rose et al., 2003). These primers were used to amplify a 172 nt fragment from *Taxus* cDNA derived from suspension cultures that had been elicited with MJ for 6 h (Figure 4A). The amplified sequence displayed 94% identity at the amino acid level to the *Solanum* JAMYC bHLH region. Only one gene was cloned using this set of degenerate primers. The *Taxus* fragment was used to obtain a full-length cDNA by screening a λ cDNA library derived from MJ-elicited cells (see Materials and methods). The protein encoded by the full-length cDNA possesses 43% overall identity with the *Solanum* JAMYC, and 59% overall similarity (Figure 4A).

To clarify the relationship of these *Taxus* genes to the bHLH genes induced by JA signaling for enhanced production of secondary metabolite biosynthesis in plants (De Geyter et al., 2012), an unrooted phylogram was generated from the *Taxus* MYC and 12 other known bHLH protein sequences using the Neighbor-Joining method (Figure 4B). The *Taxus* bHLH falls into the same clade as AtMYC2, confirming the similarity between these genes. Based on the similarity to the other well-characterized JAMYC bHLH transcription factors involved in JA mediated production of secondary metabolite, we refer to this *Taxus* gene as *TcJAMYC1* for *Taxus cuspidata* jasmonate MYC1 (TcJAMYC1).

Previously characterized JA-MYC genes from tomato and *Arabidopsis* are positively regulated by MJ with increased mRNA levels upon MJ addition (Boter et al., 2004; Dombrecht et al., 2007). To determine whether *TcJAMYC1* responds to MJ application, semi-quantitative RT-PCR was performed to compare relative transcript abundance at 1 h after MJ elicitation (Figure 4C). This result indicates that there is an increase in *TcJAMYC1* transcripts after MJ elicitation, again emphasizing the resemblance between TcJAMYC1 and the *Arabidopsis* JAMYC, AtMYC2. To find other members of JAMYC family in *Taxus*, we searched for similar cDNA sequences in a recently generated 454 GS FLX *Taxus* transcriptome sequence dataset (Lenka and Walker, unpublished). Similar to the *TcJAMYC1* mRNA, *TcJAMYC2* and *TcJAMYC4* also showed MJ-induced transcript accumulation in *Taxus* culture cells as measured by quantitative PCR both at 6 and 12 h (Figure 4D). Two other partial MYC cDNAs were identified from the *Taxus* transcriptome sequencing data (TcJAMYC3 and TcJAMYC5), but, these two MYC transcripts were not inducible by MJ (data not shown). Cloning of *TcJAMYC2* and *TcJAMYC4* was carried out using sequence specific primers designed from cDNA contigs.

### TcJAMYC1 binds to pathway promoters *in vitro* and is localized to the nucleus

**Figure 5:**
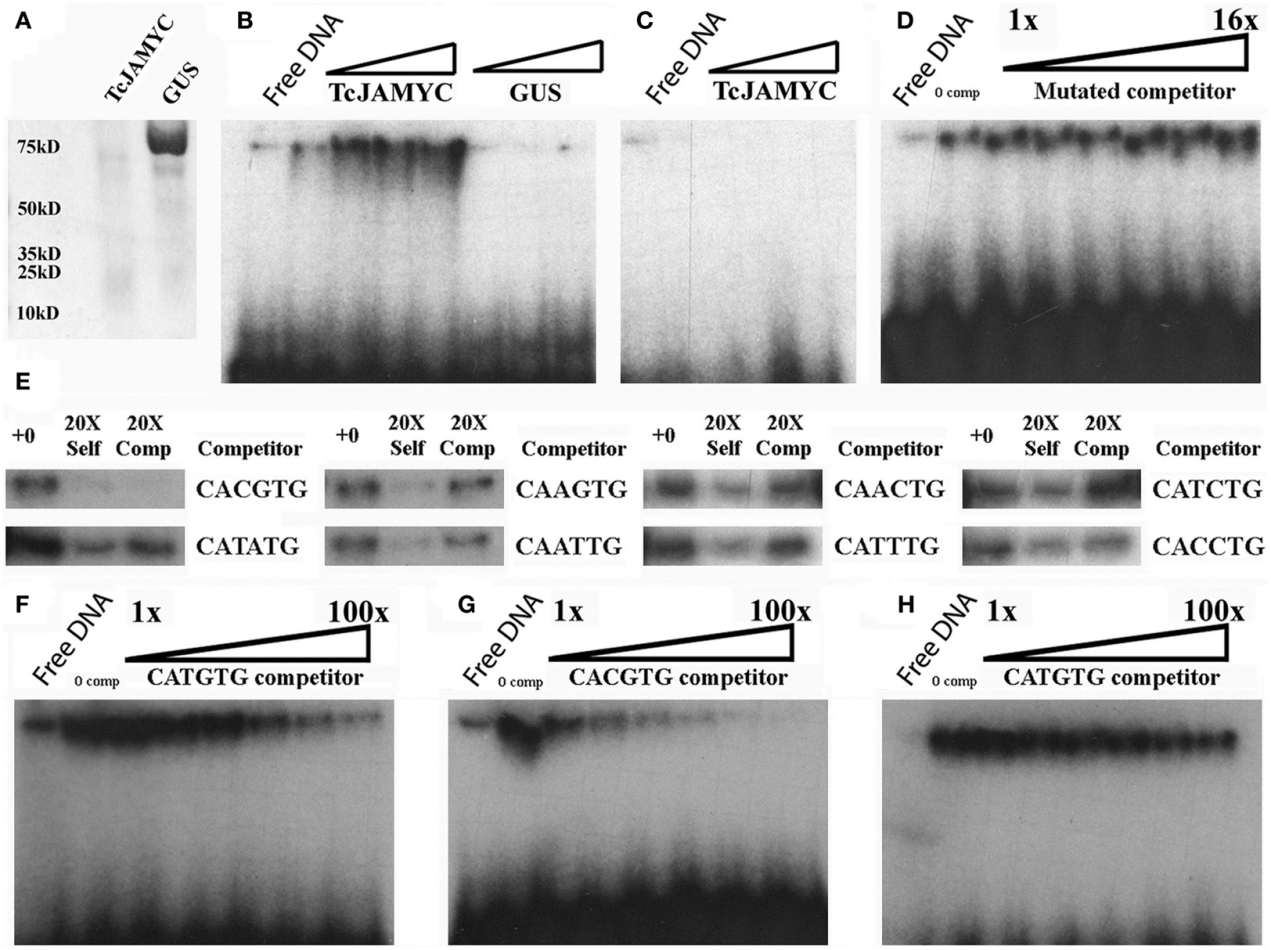
**Electrophoretic mobility shift binding assays for TcJAMYC. (A)** The TcJAMYC1 and the GUS proteins on a Coomassie blue-stained SDS page gel after nickel column purification. **(B)** EMSA using the TcJAMYC1 (left to right: ~25, 50, 75, 100 ng) and GUS (left to right: ~25, 50, and 75 ng) proteins at increasing amounts using the most common E-box element found on pathway promoters (CATGTG) as probe. **(C)** Binding assay using TcJAMYC1 at increasing amounts (left to right: ~25, 50, and 75 ng) and a mutated probe (Table 1). **(D)** Competition assay with the TcJAMYC1 protein and the CATGTG probe against increasing amounts of mutated cold competitor at 0X, 1X, 2X, 4X, 8X, and 16X excess. **(E)** Competition assays using TcJAMYC1 protein and the CATGTG radio labeled probe against all other E-box elements found in the pathway promoters. The cold competitor is listed to the right of the panels. +0: no competitor, 20X Self: the CATGTG cold competitor at 20X excess, 20X Comp: the cold competitor at 20X excess. **(F)** TcJAMYC1 self-competition assay (to determine binding affinity to CATGTG). The 1–100X range of cold competitor is 1X, 5X, 10X, 20X, 50X, and 100X excess, left to right in each gel. **(G)** A competition assay between radio labeled CATGTG against the CACGTG cold competitor. **(H)** A competition assay between radio labeled CACGTG against the CATGTG cold competitor.

To determine whether the TcJAMYC protein physically interacts with the E-box elements found in the pathway promoters, electrophoretic mobility shift assays (EMSA) were performed using one of the TF proteins TcJAMYC1. EMSAs are used to determine whether a protein can bind to a specific DNA probe sequence *in vitro*. This binding is visualized as a retarded migration rate through a native polyacrylamide gel. 6X-HIS tagged TcJAMYC1 was expressed in *E. coli* and purified on Ni-NTA agarose columns. The molecular weight for the tagged TcJAMYC1 protein was 73 kD, as expected (Figure 5A). To determine whether TcJAMYC1 binds to E-box elements, the most abundant E-box (CATGTG) in the pathway promoters was used initially as a probe sequence (Table 1). The TcJAMYC1 protein bound the CATGTG sequence (Figure 5B). To determine if the binding was specific to TcJAMYC1, the GUS protein, expressed in and purified from *E. coli* (Figure 5A), was used in the EMSA. This assay demonstrated that the binding of the CATGTG sequence is specific to the TcJAMYC1 protein, as GUS did not bind to the DNA probe (Figure 5B). Finally, a mutated DNA probe that does not contain an E-box (Table 1) was used in the EMSA (Figure 5C). The TcJAMYC1 protein did not bind to this DNA sequence, demonstrating that the TcJAMYC1 protein specifically binds to the E-box element CATGTG.

To test the specificity of binding further, a competition assay was performed using the CATGTG probe against increasing amounts of non-specific DNA competitor (the mutated DNA described above). The binding efficiency to the CATGTG probe did not diminish (Figure 5D), again demonstrating that the TcJAMYC1 protein specifically binds to the CATGTG sequence. Eight additional types of E-box sequences were found in the pathway promoters (Figure 2A). To determine whether TcJAMYC1 also binds these E-boxes, we tested oligonucleotides containing seven of these sequences in competition assays against the CATGTG probe. The binding efficiency of TcJAMYC1 toward the CAGCTG E-box element was not examined. As shown in Figure 5E, the only E-box element that competed well-against the CATGTG probe was the CACGTG element. This demonstrates that the TcJAMYC1 protein preferentially binds to two specific E-box sequences found in the pathway gene promoters: CACGTG and the CATGTG.

To test which of these two sequences, CATGTG or CACGTG, is most efficiently bound by TcJAMYC1, competition assays between these two DNA elements were performed. The CATGTG probe competed against itself, as previously shown in Figure 5E, and this was used as reference for binding specificity (Figure 5F). A competition assay was performed using the CATGTG probe against increasing amounts of CACGTG competitor (Figure 5G). The CACGTG fragment competed for binding of the CATGTG probe more effectively than CATGTG self-competitor (compare Figures 5F,G). This demonstrates that TcJAMYC1 preferentially binds to the CACGTG as compared to the CATGTG sequence. To further characterize this binding selectivity, a competition assay was performed with the CACGTG probe against the unlabeled CATGTG competitor (Figure 5H). Increasing amounts of CATGTG competitor did not efficiently compete for binding, confirming the preference for binding of CACGTG. Thus, the TcJAMYC1 protein specifically binds to CACGTG and secondarily the CATGTG E-box elements.

Nuclear localization of TcJAMYC1 was confirmed by co-localization of TcJAMYC1:GFP fusion protein in the transformed Arabidopsis protoplast nucleus (Supplementary Figures S1 A–J). This evidence suggests that TcJAMYC1 recruited to nuclear compartment might regulate pathway genes by specific interaction through the E-box elements.

### Regulation of pathway promoters by TcJAMYCs

**Figure 6:**
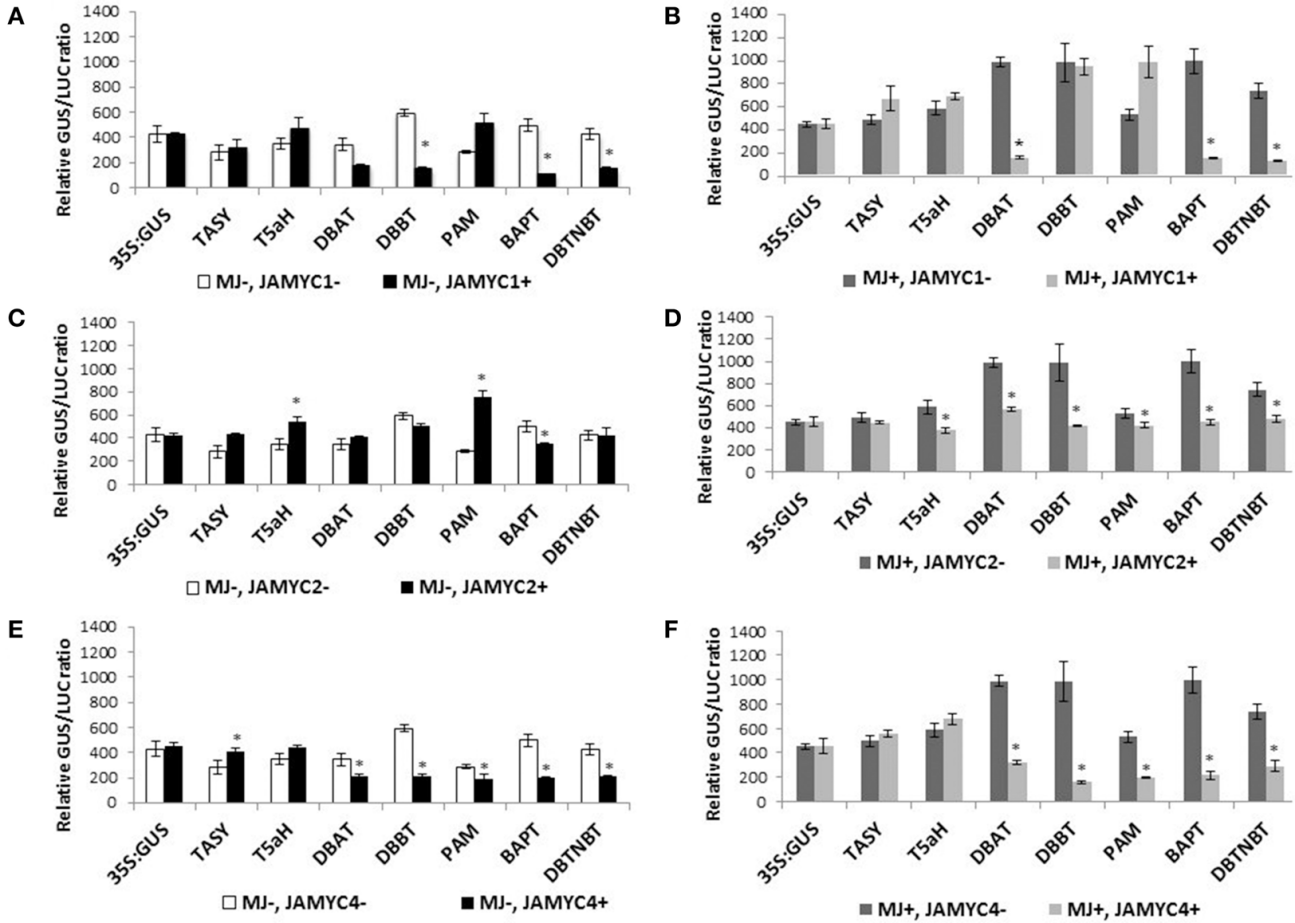
**(A–F) Promoter activation by TcJAMYC(s)**. Mock-elicited *Taxus* cultures were plated onto B5 agar plates and bombarded with the promoter:GUS fusions alone (−MJ, −MYC) or combination with the CaMV35S:TcJAMYCs effector plasmid (−MJ, +MYC) separately. MJ-elicited *Taxus* cultures were plated onto B5 agar plates and bombarded with the promoter:GUS fusions alone (+MJ, −MYC) or in combination with the CaMV35S:TcJAMYC effector plasmid (+MJ, +MYC). Results are presented as a GUS/LUC ratio. *n* = 3 in all samples, error bars are SE (^*^p < 0.05, *t*-test).

To investigate whether TcJAMYCs influence transcription of the paclitaxel pathway genes, co-bombardment experiments were performed using each of the three full-length *TcJAMYC* cDNA under the control of the CaMV35S promoter in combination with the pathway gene *promoter:GUS* reporter constructs (Figures 2A, 6A–F, Table 2). Mock-elicited cells were bombarded with each pathway gene *promoter:GUS* construct either alone or in combination with separate *35S:TcJAMYC* constructs. TcJAMYC1 negatively regulated *DBBT, BAPT*, and *DBTNBT* promoters by at least 3-fold, while all the other promoters were unaffected by co-bombardment with TcJAMYC1 (Table 2, Figure 6A). This demonstrates that TcJAMYC1 negatively regulates the promoters of the last three late pathway genes and does not affect any of the early or intermediate pathway gene promoters. On the other hand TcJAMYC2 induces the early pathway gene promoter *T5αH*, by more than 1.5-fold. TcJAMYC2 also induces *PAM* promoter by more than 2.5-fold. Expression of *BAPT* is slightly repressed by the TcJAMYC2 action and regulation of *TASY, DBAT, DBBT*, and *DBTNBT* promoters were not significantly influenced by transient over-expression of TcJAMYC2 in *Taxus* cultured cells (Table 2, Figure 6C). TcJAMYC4 has not much regulatory impact on the *T5αH* gene promoter*;* while all other pathway promoters were down regulated by TcJAMYC4 transient over-expression except *TASY*, which was weakly induced (Table 2, Figure 6E).

**Table 2:** **Changes in promoter activity caused by co-expression of TcJAMYC TF**.

**Promoters**	**Mock elicitation**			**MJ-elicited**			**E-boxes**
	**+TcJAMYC1**	**+TcJAMYC2**	**+TcJAMYC4**	**+TcJAMYC1**	**+TcJAMYC2**	**+TcJAMYC4**	
**TASY**	No change	No change	Induced (1.46-fold)	No change	No change	No change	B
**T5aH**	No change	Induced (1.55-fold)	No change	No change	Repressed (0.63-fold)	No change	D, Cx2, F
**DBAT**	No change	No change	Repressed (0.61-fold)	Repressed (0.16-fold)	Repressed (0.57-fold)	Repressed (0.31-fold)	A, G
**DBBT**	Repressed (0.25-fold)	No change	Repressed (0.35-fold)	No change	Repressed (0.42-fold)	Repressed (0.16-fold)	None
**PAM**	No change	Induced (2.62-fold)	Repressed (0.66-fold)	No change	Repressed (0.79-fold)	Repressed (0.36-fold)	Ax2, C
**BAPT**	Repressed (0.21-fold)	Repressed (0.7-fold)	Repressed (0.40-fold)	Repressed (0.15-fold)	Repressed (0.45-fold)	Repressed (0.21-fold)	H, E
**DBTNBT**	Repressed (0.36-fold)	No change	Repressed (0.50-fold)	Repressed (0.18-fold)	Repressed (0.64-fold)	Repressed (0.29-fold)	A, C, Ex2

*Expression in the presence and absence of the TF were compared separately for elicited and unelicited cells. Also shown are the types of E-boxes present within the first 250 bp of upstream sequence for each gene*.

To determine the effect of MJ elicitation on the promoter-TcJAMYC interaction, co-bombardment experiments were performed using cells that had been elicited with 100 μm MJ 6 h prior to bombardment. The elicited cells were bombarded with each *promoter:GUS* construct alone or in combination with the separate *35S:TcJAMYCs*. The *DBAT, BAPT*, and *DBTNBT* promoters were highly repressed following co-bombardment with the *35S:TcJAMYC1* effector despite MJ elicitation (Table 2, Figure 6B). The initial two pathway genes, *TASY, T5αH*, and *PAM* along with the *DBBT* promoter were not significantly regulated with MJ elicitation when co-bombarded with the *35S:TcJAMYC1* effector (Table 2, Figure 6B). However, transient over-expression of TcJAMYC2 led to a decrease in activity of all the pathway promoters when MJ elicitation was applied prior to bombardment, except *TASY*, which was unaffected (Table 2, Figure 6D). Similarly, TcJAMYC4 also negatively regulated all the pathway promoters except the first two early pathway gene promoters *TASY* and *T5αH*, whose activity remain unchanged (Table 2, Figure 6F). Thus, expression of single TcJAMYCs in elicited cells most often repressed pathway gene expression.

## Discussion

### Transcriptional activation of pathway promoters

MJ has been shown to induce terpene production in conifers (Hudgins et al., 2004), glucosinolates in *Arabidopsis* (Mikkelsen et al., 2003; Mewis et al., 2006), alkaloids in *Papaver* (Facchini and Park, 2003), *Catharanthus* (Menke et al., 1999), and *Nicotiana* (Shoji et al., 2000), as well as proteinase inhibitors in many plant species including important agricultural crops (Farmer and Ryan, 1990; Bolter, 1993; Ton et al., 2007). MJ elicitation has also been implicated in the activation of the defense related leucine aminopeptidase promoter in *Nicotiana*, the strictosidine synthase promoter in *Catharanthus*, and a sesquiterpene synthase promoter in *Nicotiana* are activated with MJ elicitation (Yin et al., 1997; Menke et al., 1999; Boter et al., 2004). Exogenously added MJ also induces the production of paclitaxel and baccatin III in *Taxus* cell suspension culture (Yukimune et al., 1996). In this investigation, we have shown that seven paclitaxel biosynthetic pathway gene promoters are likewise activated by MJ elicitation in *Taxus cuspidata* P991 suspension cell cultures. This result explains, at least in part, the previously observed increase in mRNA abundance for these genes that follows MJ elicitation (Nims et al., 2006; Li et al., 2012b), and indicates that the transcriptional response to MJ is conserved across land plant phylogeny from gymnosperms to angiosperms. The coordinated MJ inducible regulation of all the pathway promoters may indicate the presence of a common transcriptional regulatory mechanism for defense-related biosynthetic pathway genes.

### Three jasmonate-responsive bHLHs in *Taxus*

In the model plant *Arabidopsis thaliana*, MYC2 acts as a versatile regulator that is capable of both positive and negative regulation of particular pathways, including the transcriptional orchestration of other TFs (Dombrecht et al., 2007). By leveraging information about MYC2 obtained in model systems (*Arabidopsis* and *Solanum*) we have cloned TcJAMYC1, TcJAMYC2, and TcJAMYC4 from *T. cuspidata*, which produces the important pharmaceutical paclitaxel. Over-expression of particular TcJAMYCs caused positive regulation of several paclitaxel pathway gene promoters. However, most promoters (DBAT, DBBT, BAPT, and DBTNBT) were down regulated or not influenced by the individual action of the three TcJAMYC TFs (Table 2, Figures 6A–F). No strong correlation between the presence of specific E-box binding site types and the activation of particular promoters was noted. By contrast, CAGCTG was found only in those promoters (DBBT, BAPT, and DBTNBT) that were negatively regulated when co-expressed with TcJAMYC1 and TcJAMYC4 (Table 2, Figures 2A, 6A–F). Possibly, the TcJAMYCs act as heterodimers, and the effect of overexpressing a single TcJAMYC could be to disrupt the normal formation of MYC dimers, to produce a negative effect on gene expression. Alternatively, the role of TcJAMYCs in the regulation of particular paclitaxel synthesis steps may be negative. A limitation of the Taxus system is that efficient stable transformation is not yet standardized for most of the commonly used, paclitaxel-producing cell lines, such as the P991 cell line used in this study. This limits our ability to test combinations of potentially heterodimeric TcJAMYCs and to determine the effect of TcJAMYCs on the native gene activation in stably transformed cultures.

### Negative regulation of pathway promoters following MJ elicitation

*Arabidopsis thaliana* MYC2 negatively regulates some JA responsive genes and it negatively regulates its own expression (Dombrecht et al., 2007; Chico et al., 2008; Gangappa et al., 2010). In the studies presented here, we uncovered evidence for a negative regulation of the promoters of the paclitaxel biosynthetic genes by TcJAMYC proteins. In previous studies, we demonstrated that steady state mRNA levels for pathway genes increase by 6 h after MJ elicitation, remain high for about 24–30 h, and return to original low levels by about 48 h (Nims et al., 2006). This rapid up- then down-regulation of the endogenous genes indicates positive regulation for the first 24 h following elicitation, followed by negative regulation after the 24 h time point. In the studies presented here, the level of reporter gene expression was always measured 48 h after bombardment, which is ~54 h after MJ elicitation. Thus, when MJ elicitation was used, measurements were taken at a time when endogenous negative regulatory mechanisms are likely to be operative. This may help to explain the relatively weak levels of promoter activation (1.6- to 2.8-fold) that we observed from all the reporter constructs as opposed to the very strong enhancement of steady state RNA observed in RNA gel blot (Nims et al., 2006) and RNA-seq experiments (3.3- to 6.8-fold) (Li et al., 2012b). The measurements were made at this relatively late time because, following transformation by bombardment, sufficient time must be provided to allow transcription and translation of both the effector construct (TcJAMYCs) and the reporter constructs. Furthermore, reporter activity was not consistent at time points prior to 48 h post-bombardment, preventing use of earlier time points for measurements.

In no case did expression of a TcJAMYC cause enhanced reporter expression in MJ-elicited cells. Indeed, in most cases, reporter gene expression was negatively impacted by co-expression of a TcJAMYC. The most straightforward interpretation of this result is that TcJAMYCs are involved in the negative regulation of pathway genes that occurs 24–48 h after MJ elicitation. Possibly, when TcJAMYCs are overexpressed in MJ-elicited cells, negative regulation is enhanced, and lower reporter gene expression is observed. Although this is not the desired result from a metabolic engineering perspective, it could be an indication that TcJAMYCs directly regulate pathway promoters, presumably through binding to E-box elements they contain. Further efforts to introduce silencing of TcJAMYC1, 2, and 4 by MJ-inducible RNAi expression may knock down its negative regulation on late pathway genes and thus, may enable increased late taxane accumulation. An alternative explanation could be that TcJAMYCs positively regulate TFs that in turn negatively regulate pathway promoters, in much the same way that *Arabidopsis* MYC2 regulates expression of ERF11, At1g33760, and WRKY26 to impart negative regulation on several promoters (Dombrecht et al., 2007). In this scenario, overexpression of TcJAMYCs stimulates further the natural down-regulation of pathway genes that follows their initial activation by MJ, and leads to the observed decrease in reporter gene expression. Despite the seemingly conflicting or minimal effects from individual MYC proteins, it is evident that the MYC transcription factors directly bind to and affect, albeit negatively, the activation of promoters driving expression of this important medicinal plant biosynthetic pathway.

### Other transcription factors potentially regulating MJ-responsive *Taxus* genes

MJ-mediated transcriptional regulation of entire secondary pathways is not likely to be orchestrated by the action of a single TF. Combinatorial action of AP2–ERF and bHLH factors has already been shown in the JA mediated elicitation of nicotine and alkaloid biosynthesis (Zhang et al., 2011; De Geyter et al., 2012). Several other classes of TFs such as WRKYs and MYBs also have been shown to regulate JA-induced responses (Fonseca et al., 2009). Recently, an MJ-inducible WRKY transcription factor involved in promoter activation of the paclitaxel pathway gene encoding DBAT was described (Li et al., 2012a,b). The biosynthetic gene promoters described here contain putative WRKY binding sites (TGAC) on both the plus and minus strands, although not a frequencies higher than expected by chance. Thus, these promoters may be regulated by this transcription factor. Discovering the key TFs uncoupled from negative regulators and capable of partially mimicking MJ activity by activating expression of specific sets of genes is a key challenge for biologically sustainable metabolite production.

### Conflict of interest statement

The authors declare that the research was conducted in the absence of any commercial or financial relationships that could be construed as a potential conflict of interest.

## Supplementary material

Supplementary Figure S1 **Sub-cellular localization of TcJAMYC1**. 35S::*TcJAMYC1::GFP* fusion construct was transiently expressed in intact Arabidopsis mesophyll protoplasts and were imaged by a Zeiss 510 Meta laser scanning confocal microscope.
