# Supplementary material for: Jasmonate-responsive expression of paclitaxel biosynthesis genes in Taxus cuspidata cultured cells is negatively regulated by the bHLH transcription factors TcJAMYC1, TcJAMYC2, and TcJAMYC4
Source: Front Plant Sci. 2015 Feb 26;6:115. doi: 10.3389/fpls.2015.00115 (PMC4341510; doi:10.3389/fpls.2015.00115)
Supplement: Supplementary Figure S1 — Sub-cellular localization of TcJAMYC1. 35S::TcJAMYC1::GFP fusion construct was transiently expressed in intact Arabidopsis mesophyll protoplasts and were imaged by a Zeiss 510 Meta laser scanning confocal microscope. [file Presentation1.PPT]

## Slide 1
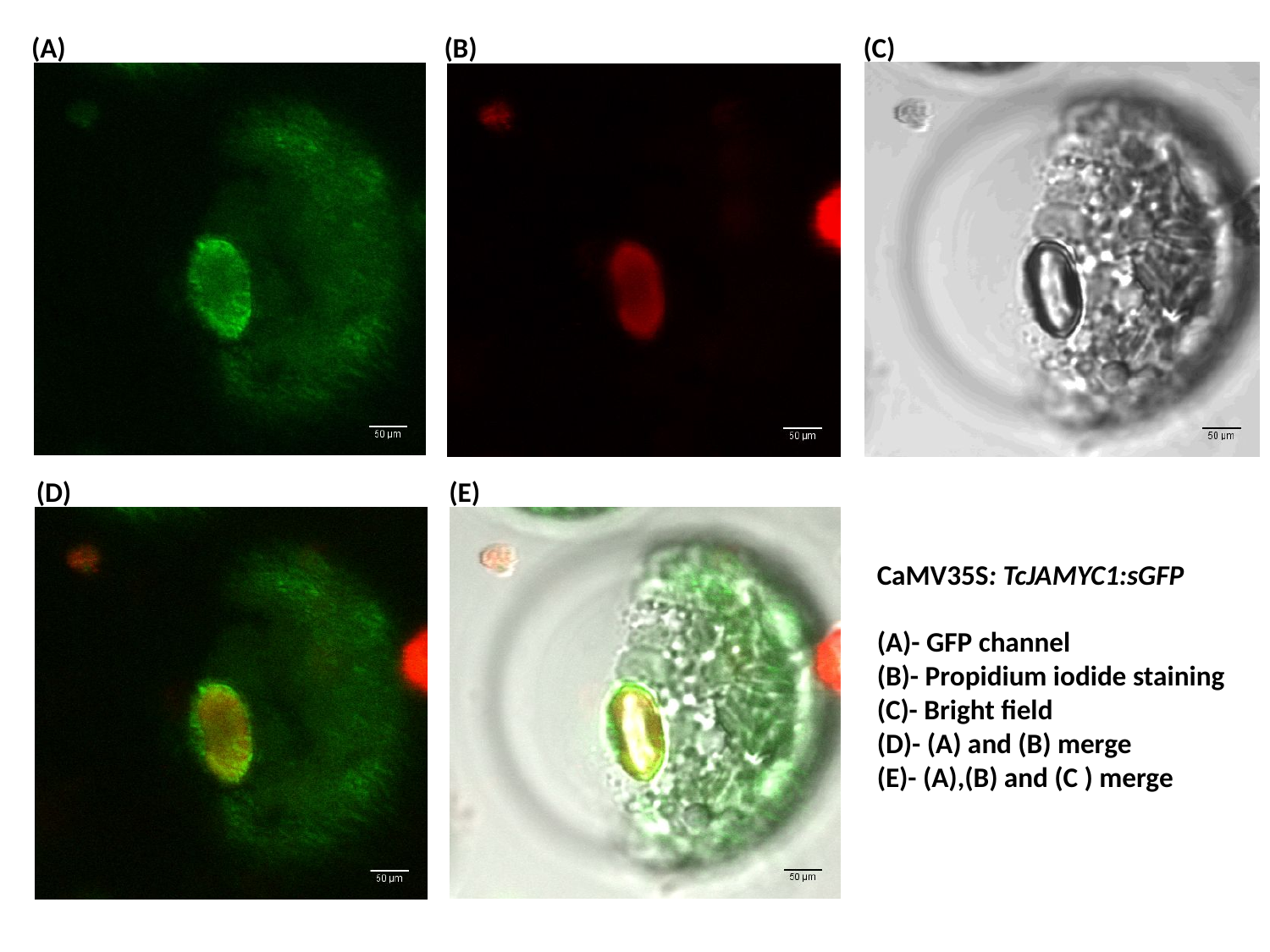

(A)			 (B)			 (C)
(D)			 (E)
CaMV35S: TcJAMYC1:sGFP
(A)- GFP channel
(B)- Propidium iodide staining
(C)- Bright field
(D)- (A) and (B) merge
(E)- (A),(B) and (C ) merge

## Slide 2
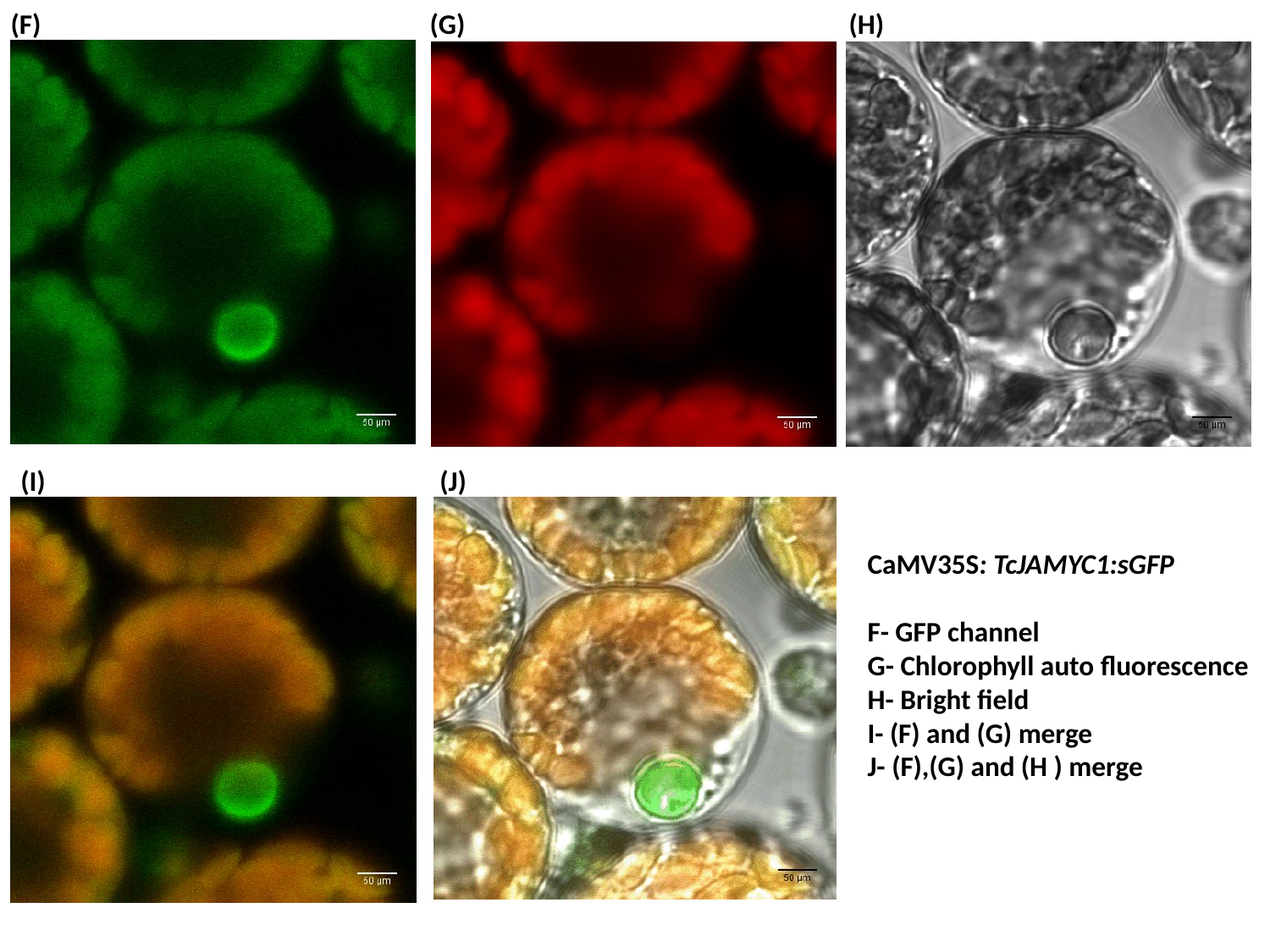

(F)			 (G)			 (H)
(I)			 (J)
CaMV35S: TcJAMYC1:sGFP
F- GFP channel
G- Chlorophyll auto fluorescence
H- Bright field
I- (F) and (G) merge
J- (F),(G) and (H ) merge
